# Network-based characterization of drug-protein interaction signatures with a space-efficient approach

**DOI:** 10.1186/s12918-019-0691-1

**Published:** 2019-04-05

**Authors:** Yasuo Tabei, Masaaki Kotera, Ryusuke Sawada, Yoshihiro Yamanishi

**Affiliations:** 1 RIKEN Center for Advanced Intelligence Project, Nihonbashi 1-chome Mitsui Building, 15th floor, 1-4-1 Nihonbashi, Chuo-ku, Tokyo, 103-0027 Japan; 2 School of Engineering, Department of Chemical System Engineering, The University of Tokyo, 7-3-1 Hongo, Bunkyo-ku, Tokyo, 113-8656 Japan; 3 Department of Bioscience and Bioinformatics, Faculty of Computer Science and Systems Engineering, Kyushu Institute of Technology, 680-4 Kawazu, Lizuka, Fukuoka, 820-8502 Japan; 4 PRESTO, Japan Science and Technology Agency, Saitama, 332-0012 Japan

**Keywords:** Drug-protein interaction prediction, Drug discovery, Large-scale prediction

## Abstract

**Background:**

Characterization of drug-protein interaction networks with biological features has recently become challenging in recent pharmaceutical science toward a better understanding of polypharmacology.

**Results:**

We present a novel method for systematic analyses of the underlying features characteristic of drug-protein interaction networks, which we call “drug-protein interaction signatures” from the integration of large-scale heterogeneous data of drugs and proteins. We develop a new efficient algorithm for extracting informative drug-protein interaction signatures from the integration of large-scale heterogeneous data of drugs and proteins, which is made possible by space-efficient representations for fingerprints of drug-protein pairs and sparsity-induced classifiers.

**Conclusions:**

Our method infers a set of drug-protein interaction signatures consisting of the associations between drug chemical substructures, adverse drug reactions, protein domains, biological pathways, and pathway modules. We argue the these signatures are biologically meaningful and useful for predicting unknown drug-protein interactions and are expected to contribute to rational drug design.

## Background

Target proteins of drug molecules are classified into a primary target and off-targets. The former is the desired target, whereas the latter could lead to adverse drug reactions [1] or unexpected beneficial effects in drug repositioning [2]. Therefore, comprehensive analysis throughout primary targets and off-targets on a genome-wide scale is crucial in drug discovery. The in silico approach is expected to improve the research productivity in this field.

Several computational methods have been presented for predicting drug-protein interactions (or compound-protein interactions) from chemogenomic and pharmacogenomic viewpoints on a large-scale. The basic idea behind the chemogenomic approach is that chemically similar drugs are expected to interact with similar proteins, with which the similarity of drugs and proteins are defined based on their side-effects and the amino acid sequences, respectively [3–8]. On the other hand, the key idea behind the pharmacogenomic approach is that phenotypically similar drugs are predicted to interact with similar proteins, on the basis of drug side effects and/or protein sequences [9–12]. However, previous predictive models are not easily interpretable, making it difficult to extract biological features characterizing drug-protein interactions and making it impossible to give insights into the theoretical basis of interactions.

The characterization of drug-protein interaction networks with biological characteristics has become a challenging problem in modern pharmaceutical science toward better understanding of poly-pharmacology. It is hypothesized that polypharmacology is involved in various features of drugs and target proteins (e.g., chemical substructures, pharmacophores, functional sites, and pathways) and complicated associations between the heterogeneous features.

A variety of feature extraction methods have recently been proposed for automatically characterizing drug-protein interactions. A data mining method was proposed for extracting molecular substructure pairs appearing frequently in interacting drug-target pairs [13]. Machine learning methods with sparse statistical models were presented to associate protein domains with drug chemical substructures [14, 15] or with drug side effects [16]. The inference of proteins eliciting drug side effects has been reported by several groups [17, 18]. However, the scalability of these methods is very limited, and these studies were conducted from the perspective of either protein functional sites, drug chemical substructures or drug phenotypic effects. There is a strong and growing need to develop efficient and scalable methods for characterizing overall drug-protein interactions with many types of features of drugs and proteins at once.

We present a novel method for systematic analyses of the underlying features characteristic of drug-protein interaction networks, which we call “drug-protein interaction signatures”. We develop a new efficient algorithm for extracting informative drug-protein interaction signatures from the integration of large-scale heterogeneous data of drugs and proteins, which is made possible by space-efficient representations for fingerprints of drug-protein pairs and sparsity-induced classifiers. In the results, our method infers a set of drug-protein interaction signatures consisting of the associations between drug chemical substructures, adverse drug reactions, protein domains, biological pathways, and pathway modules. We argue that these signatures are biologically meaningful and useful for predicting unknown drug-protein interactions. To the best of our knowledge, this is the first report on characterizing a large-scale drug-protein interaction network with various biological features of drugs and proteins in an integrative framework. The drug-protein interaction signatures comprehensively inferred with our method are expected to contribute to rational drug design.

## Results

### Drug-protein interactions

We got the information on drug-protein interactions from five databases: ChEMBL [19], KEGG [20], DrugBank [21], PDSP Ki [22], and Matador [23]. The number of unique drug-protein interactions in the merged dataset is 78,692. These interactions involve 2302 drugs and 2334 target proteins, and the number of all possible drug-protein pairs is 5,372,868. We utilized this dataset in our experiments.

### Drug profiles

We described drug chemical structures by 17,017 chemical substructures using the KEGG Chemical Function and Substructures (KCF-S) descriptor [24]. We represented each drug by a 17,017-dimension binary vector where the presence or absence of each of the KCF-S substructures is coded as 1 or 0. The resulting vector is referred to as a *chemical profile*.

We obtained the information about adverse drug reactions (ADRs) from the public release of the adverse event reporting system (AERS) of the US Food and Drug Administration (FDA) [25]. We derived 2,904,050 reports from 2004 to 2010 and mapped the drug names to KEGG following a previous study [12]. Based on the resulting 10,543 ADRs, we represented each drug by a 10,543-dimension binary vector where the presence or absence of each ADR is coded as 1 or 0. The resulting vector is referred to as an *ADR profile*.

Finally, we constructed an integrative feature vector of each drug by concatenating the chemical and the ADR profiles into a single one. The dimension of the resulting feature vector of each drug was 27,560.

### Protein profiles

We obtained functional domains, biological pathways, and pathway modules (compactly clustered pathways) about proteins from the KEGG [20] and the PFAM [26] databases.

Based on 2678 PFAM domains, we represented each protein by a 2678-dimension binary vector where the presence or absence of a functional domain is coded as 1 or 0. The resulting vector is referred to as *domain profile*. Based on 270 KEGG pathway maps, we represented each protein by a 270 dimension binary vector where the presence or absence of the involvement in a biological pathway is coded as 1 or 0. The resulting vector is referred to as a *pathway profile*. Based on 107 KEGG pathway modules, we represented each protein by a 107-dimension binary vector where the presence or absence of the involvement in a pathway module is coded as 1 or 0. The resulting vector is referred to as *module profile*.

Finally, we constructed an integrative feature vector of each protein by concatenating the domain, pathway, and module profiles into a single profile. The dimension of the resulting feature vector of each protein was 3055.

We address the problem of extracting features characterizing drug-protein interaction networks in the framework of supervised classification.

### Linear model for drug-protein pairs

Let *C* be a drug (or a drug candidate compound) and let *P* be a target protein (or a target candidate protein). We represent a drug-protein pair (*C,P*) as a high dimensional feature vector *Φ*(*C,P*) and present a linear function, *f*(*C,P*)=**w**^*T*^*Φ*(*C,P*), whose output is used to predict whether a (*C,P*) is an interacting pair or not. The weight vector **w** is estimated such that each drug-protein pair is correctly classified into the interaction class (positive class) or non-interaction class (negative class) based on the training set.

An advantage of the linear model is that one can interpret features effective for predictions from learned models. Since each element in *Φ*(*C,P*) corresponds to an element of **w**, effective features can be selected by extracting highly weighted features. However, the performance of the linear model depends heavily on the feature vector design.

We represent each drug-protein pair as a high dimension feature vector by taking the tensor product of a drug profile and protein profile. The representation is similar to that in previous studies [15, 16]. The profile of a *C* is defined as a *D*-dimension binary vector:

*Φ*(*C*)=(*c*_1_,*c*_2_,...,*c*_*D*_)^*T*^,

where *c*_*i*_∈{0,1}, *i*=1,...,*D*. The profile of a *P* is defined as a *D*^′^-dimension binary vector: $\Phi (P)=(p_{1},p_{2},...,p_{D^{\prime }})^{T}$, where *p*_*i*_∈{0,1}, *i*=1,...,*D*^′^. We compute the tensor product between a drug profile *Φ*(*C*) and protein profile *Φ*(*P*), and define a feature vector *Φ*(*C,P*) as follows:

$$\Phi(C,P) = (c_{1}p_{1},c_{1}p_{2},...,c_{1}p_{D^{\prime}},c_{2}p_{1},...c_{D}p_{1},...,c_{D}p_{D^{\prime}})^{T}. $$

where *Φ*(*C,P*) is composed of all possible products between elements in *Φ*(*C*) and those in *Φ*(*P*). The resulting feature vector is a *D*×*D*^′^-dimension binary vector, i.e., fingerprint, for encoding cross-integrated biological features. This is referred to as a *“tensor-product fingerprint”*.

In this study, *Φ*(*C*) was a 27,560-dimension binary vector, and *Φ*(*P*) was a 3055-dimension binary vector. Thus, the tensor-product fingerprint *Φ*(*C,P*) of each drug-protein pair is a 84,195,800-dimension binary vector.

A simpler way for representing each drug-protein pair is to concatenate *Φ*(*C*) and *Φ*(*P*) into a single feature vector as *Φ*(*C,P*)=(*Φ*(*C*)^*T*^,*Φ*(*P*)^*T*^)^*T*^ [7]. However, it cannot determine the correlation between drug and protein features. The feature vector is referred to as a *“concatenated fingerprint”*.

### Logistic regression

We apply logistic regression to train the weight vector in the linear model and introduce *L*_1_-regularizations to prevent over-fitting. The *L*_1_-regularization induces sparsity in the weight vector and drives most of the weight elements corresponding to unimportant features to zeros, which makes it easier for us to interpret the model and extract features.

Minimizing the logistic loss with *L*_1_-regularization for a large number of high dimensional data is difficult, but several efficient algorithms have recently been proposed. To the best of our knowledge, LIBLINEAR [27] is the most efficient and high-performance algorithm, but it requires a huge amount of memory for extremely high-dimensional data. In fact, it was not computationally feasible for our dataset in this study because of the memory problem (see the “Results” section). To overcome this difficulty, we introduce a *gradient-based method*.

Given a collection of drug-protein pairs and their labels (*Φ*(*C*_*i*_,*P*_*j*_),*y*_*ij*_) where *y*_*ij*_∈{+1,−1}(*i*=1,...,*n,j*=1,...,*m*), the logistic loss is defined as

$$LR(\mathbf{w}) = \sum\limits_{i=1}^{n}\sum\limits_{j=1}^{m} \log (1+\exp \left(-y_{ij}\mathbf{w}^{T}\Phi(C_{i},P_{j}) \right). $$

The logistic loss with *L*_1_-regularization is defined as

$$ L_{1}\text{-}LR(\mathbf{w}) \,=\,\! \sum\limits_{i=1}^{n}\!\sum\limits_{j=1}^{m} \!\log\! \left(\!1\,+\,\exp\! \left(\!-y_{ij}\mathbf{w}^{T}\Phi(C_{i},P_{j})\! \right) \!\right) + C\|\mathbf{w}\|_{1}, $$

where ∥**w**∥_1_ is *L*_1_ norm (the sum of absolute value in the vector) and *C* is a regularization parameter.

Since *L*_1_-*LR*(**w**) is a convex function, the weight vector **w** minimizing *L*_1_-*LR*(**w**) can be found at zero of its gradient. However, it is impossible to compute the gradient of *L*_1_-*LR*(**w**), because *L*_1_ norm contains non-differential points where *w*_*d*_=0. Instead, we compute the *d*-th dimensional gradient ∇_*d*_*LR*(**w**) of *LR*(**w**) as follows:

$$ \nabla_{d} LR(\mathbf{w}) = \sum\limits_{i=1}^{n}\sum\limits_{j=1}^{m} \frac{-y_{ij} \Phi_{d}(C_{i},P_{j}) \exp \left(-y_{ij}\mathbf{w}^{T}\Phi(C_{i},P_{j}) \right)}{1+\exp \left(-y_{ij}\mathbf{w}^{T}\Phi(C_{i},P_{j}) \right)}, $$

where *Φ*_*d*_(*C*_*i*_,*P*_*j*_) is the *d*-th dimensional value of *Φ*(*C*_*i*_,*P*_*j*_). We then compute the *D*×*D*^′^-dimensional gradient vector $\nabla LR(\mathbf {w}) \in \mathfrak {R}^{D\times D^{\prime }}$ as

$$\nabla LR(\mathbf{w}) = \left(\nabla_{1} LR(\mathbf{w}),\nabla_{2} LR(\mathbf{w}),...,\nabla_{D\times D^{\prime}} LR(\mathbf{w}) \right)^{T}. $$

The use of ∇*LR*(**w**) enables the global minimum for the optimal **w** in *L*_1_-*LR*(**w**) to be found using an efficient gradient-based optimization algorithm called orthant-wise limited-memory quasi-newton (OWL-QN) [28]. The *L*_1_-regularized logistic regression methods, with the tensor product of the fingerprint proposed and with the concatenated fingerprint, is referred to as *L1LOG-tensor* and *L1LOG-concat*, respectively.

For comparison, we also trained models with *L*_2_-regularized logistic regression using the gradient-based algorithm called the limited memory quasi-Newton (L-BFGS) [29]. The *L*_2_-regularized logistic regression method, with the tensor-product fingerprint and the concatenated fingerprint, are referred to as *L2LOG-tensor* and *L2LOG-concat*, respectively.

### Space-efficient representation of drug-protein pairs

Compact representation of drug-protein pairs is crucial for training linear models in memory, so we use the set representation with items corresponding to dimensions of one bit in the fingerprint. However, this still consumes a huge amount of memory for storing them, resulting in limited scalability in memory for extremely high-dimensional data. To overcome this memory problem, we constructed two space-efficient representations of fingerprints. We present a brief overview of these representations (further details are given in the supplemental material [30]).

**Fig. 1:**
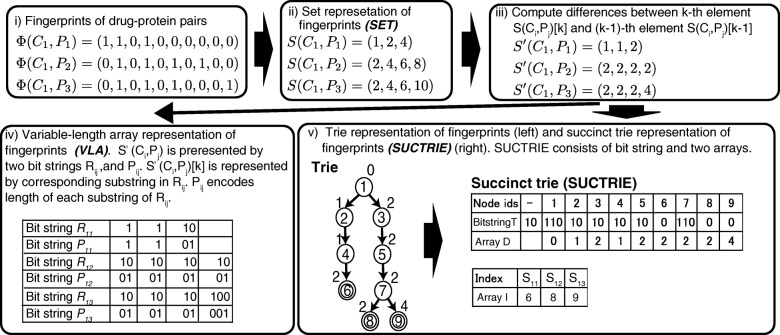
Brief summary of constructing space-efficient representations of fingerprints for drug-protein pairs constructed with our proposed method: VLA and SUCTRIE

Figure 1 illustrates the construction of our two representations. We first represent each fingerprint *Φ*(*C*_*i*_,*P*_*j*_) as a set *S*(*C*_*i*_,*P*_*j*_)={*d*|*Φ*_*d*_(*C*_*i*_,*P*_*j*_)=1,*d*=1,...,*D*×*D*^′^} that contains items corresponding to dimensions of one bit in *Φ*(*C*_*i*_,*P*_*j*_). We refer to a set representation of fingerprints as *SET*. To minimize each item, we then compute the difference between the *k*-th item *S*(*C*_*i*_,*P*_*j*_)[*k*] and (*k*−1)-th item *S*(*C*_*i*_,*P*_*j*_)[*k*−1] as (*S*(*C*_*i*_,*P*_*j*_)[*k*]−*S*(*C*_*i*_,*P*_*j*_)[*k*−1]) and keep the results in a new set *S*^′^(*C*_*i*_,*P*_*j*_). We can recover *S*(*C*_*i*_,*P*_*j*_) by cumulatively adding the items in *S*^′^(*C*_*i*_,*P*_*j*_).

We constructed our two space-efficient representations of fingerprints by leveraging the idea behind *succinct data structures* that achieve space-efficient representations of data structures while preserving the property of fast operations. The first one is a variable-length array for compactly representing fingerprints. The *S*^′^(*C*_*i*_,*P*_*j*_) is represented by two bit strings *R*_*ij*_ and *P*_*ij*_ which are indexed by *rank/select dictionary*, i.e., a succinct data structure for bit strings. We can randomly access any element in *S*^′^(*C*_*i*_,*P*_*j*_) in *O*(1) time by using fast operations in the rank/select dictionary [31]. We refer to this variable-length array representation of fingerprints as *VLA*.

The second one is a type of succinct trie for representing fingerprints. The trie is a data structure for strings, and it is also practical for representing fingerprints. A standard point-based implementation of trie consumes a huge amount of memory, resulting in limited scalability. Alternatively, we present a compact representation of trie by using a succinct data structure called LOUDS [32]. We can recover the original fingerprints by traversing a succinct trie in a depth-first manner. We refer to this succinct trie representation of fingerprints as *SUCTRIE*.

### Extraction of drug-protein interaction signatures

We applied the proposed method (L1LOG-tensor) to extract drug-protein interaction signatures from drug profiles (chemical substructures and adverse drug reactions) and protein profiles (protein domains, biological pathways, and pathway modules), based on a large-scale drug-protein interaction network. Each signature is the association between a drug feature and protein feature, where two features in the same signature are thought of as being associated in terms of drug-protein interactions. The results of all extracted drug-protein interaction signatures are presented in the supplemental material [30].

L1LOG-tensor extracted 105,684 signatures, while L2LOG-tensor extracted 7,843,218 signatures. Note that the number of all possible combinations of drug features and protein features is 84,195,504. The number of signatures from our L1LOG-tensor method was much less than that of L2LOG-tensor, due to the sparsity induced by L1-regularization. This makes it easier to analyze the extracted drug-protein interaction signatures for biological interpretation, so we focused on analyzing the results from L1LOG-tensor below.

**Fig. 2:**
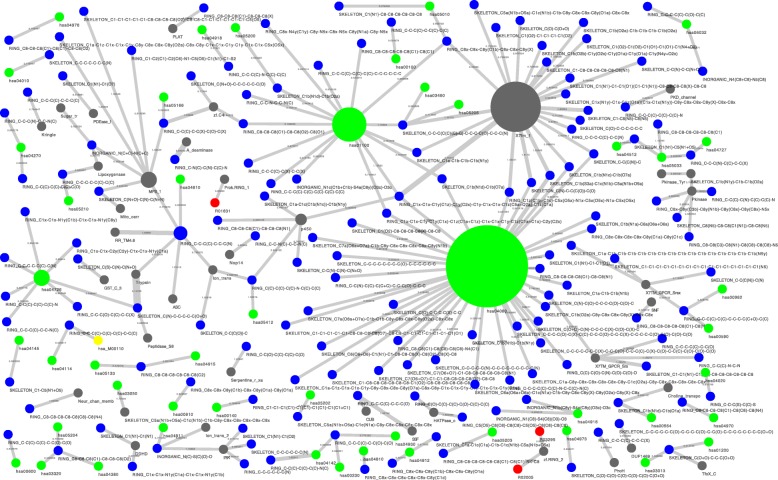
Part of obtained drug-protein interaction signature network among five features, i.e., drug chemical substructures (blue), adverse drug reactions (red), protein domain (gray), biological pathway (green), and pathway module (yellow). Node size represents degree of each feature, and edge width represents corresponding weight in model

Figure 2 shows a network representation of some of the drug-protein signatures extracted with L1LOG-tensor, where highly weighted associations of five features of drugs or proteins, that is, drug-chemical substructures (blue), adverse drug reaction (red), protein pathway (green), pathway module (yellow) and protein domain (gray). Only selected results are shown due to space limitation. The inferred signature association network provides us with clues about the important features behind the drug-protein interaction network. There has been no study on the inference of these associations.

### Biological interpretation of the extracted signatures

We constructed biological interpretations for the drug-protein interaction signatures extracted with L1LOG-tensor. We give only two examples due to space limitation. The result of all analyzed signatures and the figures/tables are presented in the supplemental material [30].

**Fig. 3:**
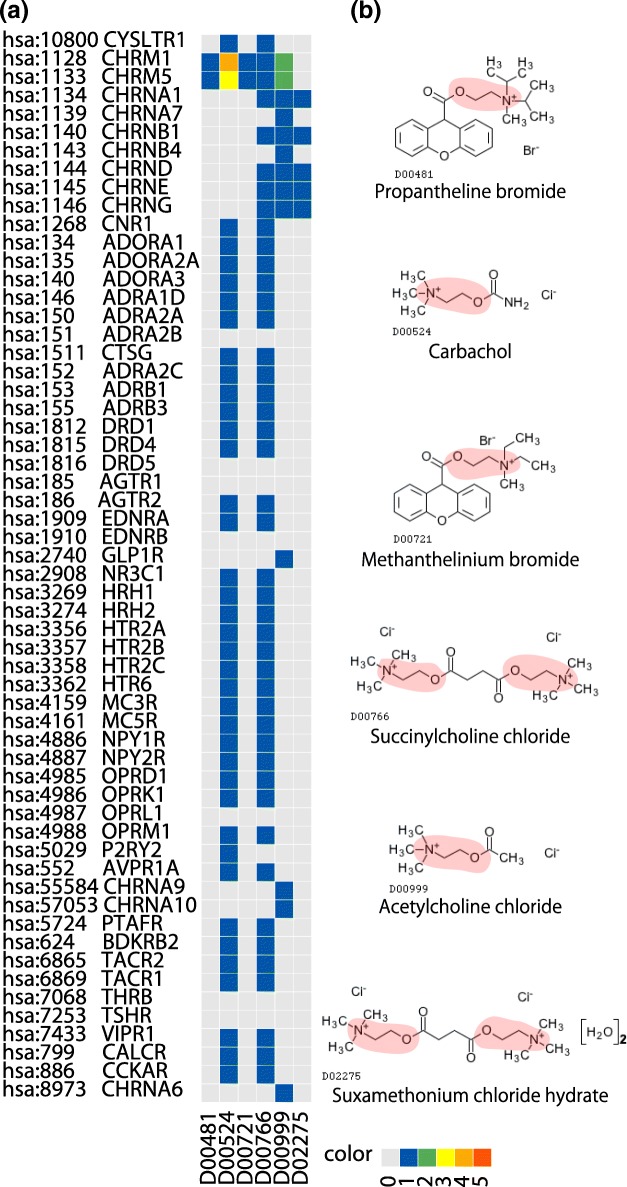
Example of drug-protein interaction signature: association between drug-chemical substructure (SKELETON C1b(N1d)-C1b(O7a) in KCF-S format) and biological pathway (hsa04080 Neuroactive ligand-receptor interaction). **a** Horizontal axis shows drugs sharing chemical substructure, and vertical axis shows proteins sharing biological pathway. Color of each element corresponds to number of databases storing corresponding interaction. **b** Chemical structures of drugs sharing substructure are shown, and extracted substructure is highlighted in red (see Table 1 for further details)

**Fig. 4:**
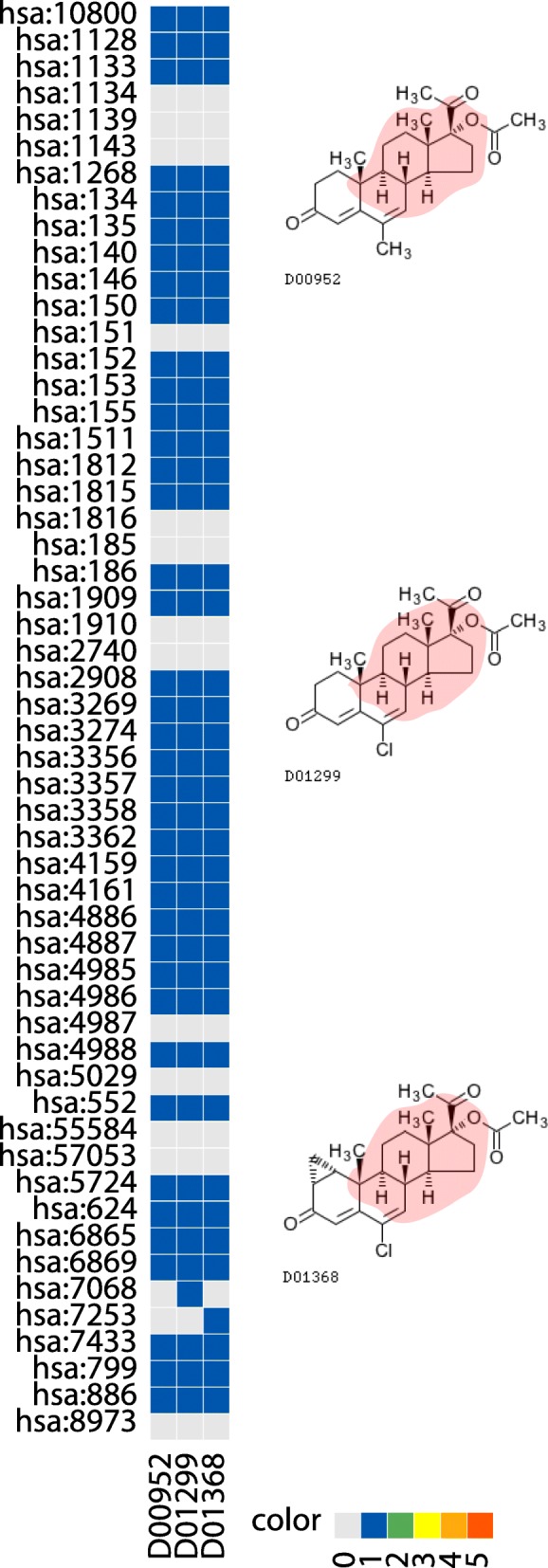
The association between KCF-S “RING C1x-C1x-C1y(C1z)-C1y(C2x)-C1y-C1x-C1x-C1z(C5a+O7a)-C1z(C1a)” and KEGG pathway hsa04080 Neuroactive “ligand-receptor interaction” **a** The heat map shows the numbers of databases that register confirmed drug-protein interactions from KEGG, DrugBank, Matador, Chembl, PSD pi databases. Horizontal and vertical axes show drugs and proteins, respectively. Gray, blue, green, yellow, orange and red indicate that 0, 1, 2, 3, 4 and 5 databases contain the corresponding interaction. **b** Chemical structures of some drugs, where red areas (if any) show the extracted substructure indicated by KCF-S. See also Table 1

Figure 3 shows an extracted signature representing the association between a drug-chemical substructure (SKELETON C1b(N1d)-C1b(O7a) in the KCF-S format) and biological pathway (hsa04080 Neuroactive ligand-receptor interaction), where the vertical axis on the heat map (a) shows all drugs sharing the extracted substructure, and the horizontal axis shows all proteins sharing the extracted pathway. The extracted drug-chemical substructures on the associated drug structures (b) are in pink. Drugs and proteins in known interacting pairs tend to have such extracted features in the same signature. For example, Propantheline bromide (D00481), Methanthelinium bromide (D00721), Acetylcholine chloride (D00999), Carbachol (D00524), Succinylcholine chloride (D00766), and Suxamethonium chloride (D02275) share a choline skeleton, and all known to act on acetylcholine receptors. However, there are many other drugs sharing the extracted drug feature and proteins sharing the extracted protein feature, and the drug-protein interactions are not known. Thus, it may be possible to predict previously unknown interactions between drugs and proteins through the extracted features in the signatures. See Table 1 and Fig. 4 for detail.

**Table 1:** Association between KCF-S “RING C1x-C1x-C1y(C1z)-C1y(C2x)-C1y-C1x-C1x-C1z(C5a+O7a)-C1z(C1a)” and KEGG pathway hsa04080 Neuroactive “ligand-receptor interaction”. See also Fig. 4

KCF-S	RING C1x-C1x-C1y(C1z)-C1y(C2x)-C1y-C1x-
	C1x-C1z(C5a+O7a)-C1z(C1a)
Pathway	hsa04080 Neuroactive ligand-receptor interaction
Drug	D00952 Megestrol acetate (antineoplastic)
	D01299 Chlormadinone acetate (progestin)
	D01368 Cyproterone acetate (anti-androgen)
Protein	hsa:10800 cysteinyl leukotriene receptor 1
	hsa:1128-hsa:1133 muscarinic acetylcholine receptor M1 - M5
	hsa:1134 nicotinic acetylcholine receptor alpha-1
	hsa:1268 cannabinoid receptor 1
	hsa:134,hsa:135,hsa:140 adenosine A1 receptor A1, A2a, A3
	hsa:146,hsa:150,hsa:151 adrenergic receptor alpha-1D,2A,2B
	hsa:1511 cathepsin G
	hsa:152 adrenergic receptor alpha-2C
	hsa:153-hsa:155 adrenergic receptor beta-1,2,3
	hsa:1812-hsa:1816 dopamine receptor D1-D5
	hsa:185-hsa:186 angiotensin II receptor type 1,2
	hsa:1909-hsa:1910 endothelin receptor type A, B
	hsa:2908 glucocorticoid receptor
	hsa:3269,hsa:3274 histamine receptor H1,H2
	hsa:3356,hsa:3357,hsa:3358 5-hydroxytryptamine receptor 2
	hsa:3362 5-hydroxytryptamine receptor 6
	hsa:4159-hsa:4161 melanocortin receptor 3,4,5
	hsa:4886,hsa:4887 neuropeptide Y receptor type 1/4/6,2
	hsa:4985 delta-type opioid receptor
	hsa:4986 kappa-type opioid receptor
	hsa:4988 mu-type opioid receptor
	hsa:552 arginine vasopressin receptor 1A
	hsa:5724 platelet-activating factor receptor
	hsa:624 bradykinin receptor B2
	hsa:6865,hsa:6869 tachykinin receptor 1,2
	hsa:7068 thyroid hormone receptor beta
	hsa:7253 thyroid stimulating hormone receptor
	hsa:7433 vasoactive intestinal peptide receptor 1
	hsa:886 cholecystokinin A receptor

**Fig. 5:**
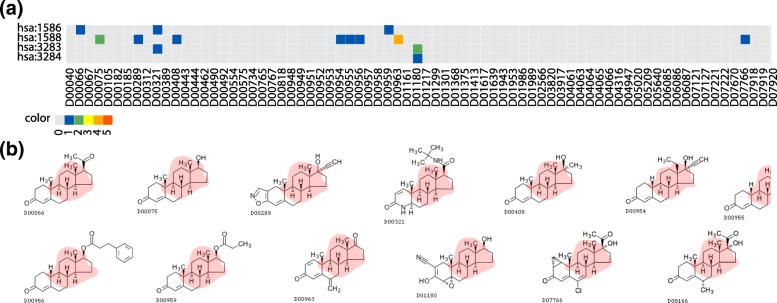
The association between KCF-S “RING C1x-C1x-C1y(C1z)-C1y(C1x)-C1y(C1x)-C1z(C1a+C1y)” and KEGG pathway module “hsa_M00110 C19/C18-Steroid hormone biosynthesis”. **a** The heat map shows the numbers of databases that register confirmed drug-protein interactions from KEGG, DrugBank, Matador, Chembl, PSD pi databases. Horizontal and vertical axes show drugs and proteins, respectively. Gray, blue, green, yellow, orange and red indicate that 0, 1, 2, 3, 4 and 5 databases contain the corresponding interaction. **b** Chemical structures of some drugs, where red areas (if any) show the extracted substructure indicated by KCF-S. See also Table 3

Table 2 shows an extracted signature representing the association between an ADR (R01631 Graft-versus-host disease) and protein domain (PF14446 Prokaryotic RING finger family 1), where all drugs sharing the extracted ADR and all proteins sharing the extracted protein domain are also shown. Interestingly, most drugs sharing the ADR (R01631 Graft-versus-host disease) were related to inflammation, immunosuppression, and cancer, which supports the recently expanded concept that inflammation is a critical component of cancer progression [33]. See Fig. 5 and Table 3 for detail.

**Table 2:** Example of drug-protein interaction signature: association between adverse drug reaction (ADR) (R01631 Graft-versus-host disease) and protein domain (PF14446 Prokaryotic RING finger family 1)

Extracted ADR	R01631 Graft-versus-host disease
Extracted domain	PF14446 Prokaryotic RING finger family 1
Drugs sharing the extracted ADR	D00322 Fluconazole (antifungal)
	D00333 Ganciclovir (antiviral)
	D00399 Valproic acid (anticonvulsant)
	D00407 Methylprednisolone (glucocorticoid)
	D06272 Sorafenib tosilate (anticancer, antineoplastic)
	D06413 Nilotinib hydrochloride (antineoplastic)
	D08062 Idarubicin (antineoplastic, antibiotic)
	D08066 Imatinib (antineoplastic)
	D08524 Sorafenib (antineoplastic, anticancer)
	D08556 Tacrolimus (immunosuppressant)
Proteins sharing extracted domain	hsa:5587,hsa:23683,hsa:25865 protein kinase D
	hsa:51317 PHD finger protein 21A
	hsa:5580 protein kinase C
	hsa:64283 Rho guanine nucleotide exchange factor
	hsa:673 B-Raf proto-oncogene protein kinase
	hsa:80829 ZFP91 zinc finger protein

Further details are given in Fig. 5

**Table 3:** The association between KCF-S “RING C1x-C1x-C1y(C1z)-C1y(C1x)-C1y(C1x)-C1z(C1a+C1y)” and KEGG pathway module “hsa_M00110 C19/C18-Steroid hormone biosynthesis”

KCF-S	RING C1x-C1x-C1y(C1z)-C1y(C1x)-C1y(C1x)-C1z(C1a+C1y)	
Module	hsa_M00110 C19/C18-Steroid hormone biosynthesis	
Drug	D00040 Cholesterol (pharmaceutic aid)	D00066 Progesterone (progestin)
	D00067 Estrone (estrogen)	D00075 Testosterone (androgen)
	D00105 Estradiol (estrogen)	D00182 Norethisterone (progestin)
	D00185 Estriol (estrogen)	D00289 Danazol (Anterior pituitary suppressant)
	D00312 Estrone sodium sulfate (estrogen)	D00321 Finasteride (alpha-reductase inhibitor)
	D00389 Metandienone (androgen)	D00408 Methyltestosterone (androgen)
	D00443 Spironolactone (diuretic)	D00444 Stanozolol (androgen)
	D00462 Oxandrolone (androgen)	D00490 Oxymetholone (androgen)
	D00492 Pancuronium (neuromuscular blocking agent)	D00554 Ethinylestradiol (estrogen)
	D00575 Mestranol (estrogen)	D00734 Ursodeoxycholic acid (anticholelithogenic)
	D00765 Rocuronium (neuromuscular blocking agent)	D00767 Vecuronium (neuromuscular blocking agent)
	D00818 Maprotiline hydrochloride (antidepressant)	D00948 Estropipate (estrogen)
	D00949 Hydroxyprogesterone caproate (progestin)	D00951 Medroxyprogesterone acetate (progestin)
	D00952 Megestrol acetate (antineoplastic)	D00953 Norethisterone acetate (progestin)
	D00954 Norgestrel (progestin)	D00955 Nandrolone decanoate (androgen)
	D00956 Nandrolone phenylpropionate (androgen)	D00957 Testosterone cypionate (androgen)
	D00958 Testosterone enanthate (androgen)	D00959 Testosterone propionate (androgen)
	D00963 Exemestane (antineoplastic)	D01161 Fulvestrant (antiestrogen)
	D01180 Trilostane (adrenocortical suppressant)	D01217 Dydrogesterone (progestin)
	D01299 Chlormadinone acetate (progestin)	D01301 Metenolone enanthate (anabolic)
	D01368 Cyproterone acetate (anti-androgen)	D01375 Metenolone acetate (anabolic)
	D01413 Estradiol valerate (estrogen)	D01617 Estradiol dipropionate (estrogen)
	D01639 Tibolone (Menopausal symptoms suppressant)	D01943 Potassium canrenoate (aldosterone antagonist)
	D01953 Estradiol benzoate (estrogen)	D01986 Estriol tripropionate (estrogen)
	D01989 Estriol diacetate benzoate (estrogen)	D02566 Maprotiline (antidepresant)
	D03820 Dutasteride (prostatic hyperplasia)	D03917 Drospirenone (aldosterone antagonist)
	D04061 Estradiol acetate (estrogen)	D04063 Estradiol cypionate (estrogen)
	D04064 Estradiol enanthate (estrogen)	D04065 Estradiol undecylate (estrogen)
	D04066 Estramustine (antineoplastic)	D04316 Gestodene (progestin)
	D04947 Mesterolone (androgen)	D05020 Mexrenoate potassium (aldosterone antagonist)
	D05209 Norgestimate (progestin)	D05640 Prorenoate potassium (aldosterone antagonist)
	D06085 Testosterone ketolaurate (androgen)	D06086 Testosterone phenylacetate (androgen)
	D06087 Testosterone undecanoate (testosterone)	D07121 Alfatradiol (five alfa-reductase inhibitor)
	D07127 Norethandrolone (anabolic)	D07221 Promestriene (estrogen)
	D07222 Nomegestrol (progestin)	D07670 Chlormadinone (progestin)
	D07766 Cyproterone (antiandrogen)	D07918 Estradiol hemihydrate (estrogen)
	D07919 Estradiol 17 beta-hemisuccinate (estrogen)	D07920 Estriol succinate (estrogen)
	D07921 Estriol sodium succinate (estrogen)	D08052 Hydroxyprogesterone (progestin)
	D08166 Medroxyprogesterone (progestin, antineoplastic)	D08167 Megestrol (progestin)
	D08250 Nandrolone (anabolic, ophthalmic)	D08281 Nomegestrol acetate (contraceptive)
	D08285 Norethisterone enantate (progestin)	D08409 Prasterone (androgen)
	D08573 Testosterone decanoate (androgen)	D08574 Testosterone phenylpropionate (androgen)
	D09701 Abiraterone acetate (anticancer)	
Protein	hsa:1586 cytochrome P450, family 17, subfamily A	
	hsa:1588 cytochrome P450, family 19, subfamily A	
	hsa:3283 steroid delta-isomerase	
	hsa:3284 steroid delta-isomerase	

See also Fig. 5

Figure 4 shows an extracted signature representing the association between a drug-chemical substructure (RING C1x-C1x-C1y(C1z)-C1y(C2x)-C1y-C1x-C1x-C1z(C5a+O7a)-C1z(C1a) in the KCF-S format) and biological pathway (hsa04080 Neuroactive ligand-receptor interaction). It was observed that Megestrol acetate (D00952), Cyproterone acetate (D01368) and Chlormadinone acetate (D01299) share common ring structures. All these drugs are known to act on many neuroactive ligand-receptors. See Table 1 for detail.

**Fig. 6:**
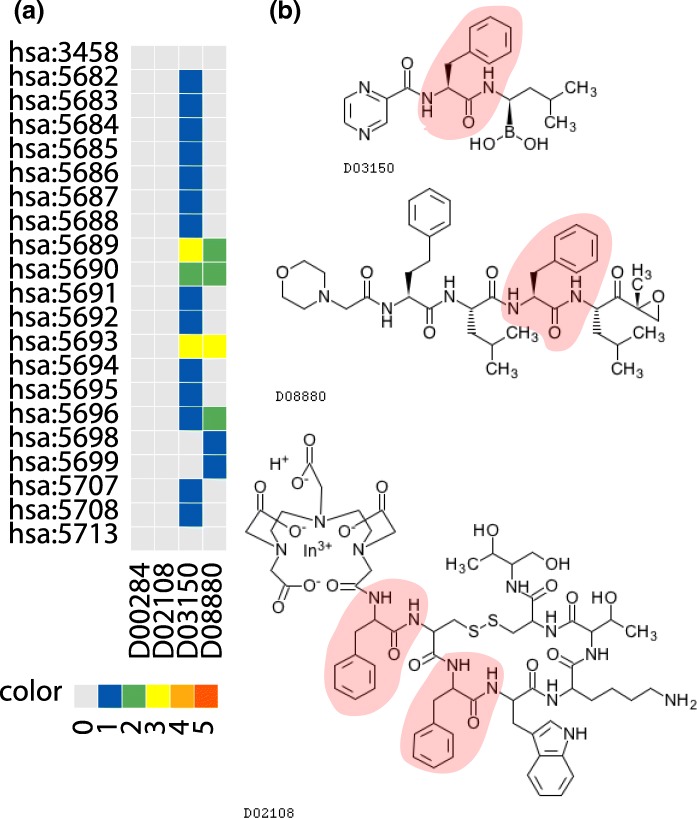
The association between KCF-S “SKELETON C5a(N1b+O5a)-C1c(N1b)-C1b-C8y-C8x-C8x-C8x-C8x-C8x” and KEGG pathway “hsa03050 Proteasome” **a** The heat map shows the numbers of databases that register confirmed drug-protein interactions from KEGG, DrugBank, Matador, Chembl, PSD pi databases. Horizontal and vertical axes show drugs and proteins, respectively. Gray, blue, green, yellow, orange and red indicate that 0, 1, 2, 3, 4 and 5 databases contain the corresponding interaction. **b** Chemical structures of some drugs, where red areas (if any) show the extracted substructure indicated by KCF-S. See also Table 4 for detail

Figure 6 show an extracted signature representing the association between a drug-chemical substructure (SKELETON C5a(N1b+O5a)-C1c(N1b)-C1b-C8y-C8x-C8x-C8x-C8x-C8x in the KCF-S format) and biological pathway (hsa03050 Proteasome). Proteasome inhibitors have been applied to the treatment of cancer, especially multiple myeloma. The substructure “SKELETON C5a(N1b+O5a)-C1c(N1b)-C1b-C8y-C8x-C8x-C8x-C8x-C8x” corresponds to a phenylalanine residue, which is captured as a characteristic substructure in known proteasome inhibitors Bortezomib (D03150) and Carfilzomib (D08880). See Table 4 for detail.

**Table 4:** The association between KCF-S “SKELETON C5a(N1b+O5a)-C1c(N1b)-C1b-C8y-C8x-C8x-C8x-C8x-C8x” and KEGG pathway “hsa03050 Proteasome”

KCF-S	SKELETON C5a(N1b+O5a)-C1c(N1b)-C1b-C8y-C8x-
	C8x-C8x-C8x-C8x
Pathway	hsa03050 Proteasome
Drug	D00284 Cosyntropin (hormone, adrenocorticotropic)
	D02108 Indium In 111 pentetreotide (radioactive agent)
	D03150 Bortezomib (anticancer, proteasome inhibitor)
	D08880 Carfilzomib (anticancer, proteasome inhibitor)
Protein	hsa:3458 interferon gamma
	hsa:5682 - hsa:5688 20S proteasome subunit alpha 1-7
	hsa:5689 - hsa:5699 20S proteasome subunit beta 1-10
	hsa:5707, hsa:5708, hsa:5713 26S proteasome regulatory subunit N1, N2, N8

See also Fig. 6

### Performance evaluation on generalization property

If the extracted signatures are biologically meaningful in terms of drug-protein interactions, they need to have good generalization to predict drug-protein interactions.

We tested five feature extraction methods: L1LOG-tensor, L2LOG-tensor, L1LOG-concat, L2LOG-concat, and L1LOG-LIBLINEAR-tensor on their abilities to reconstruct known drug-protein interactions. As mentioned above, L1LOG-tensor is our proposed method. The others are previous methods based on current algorithms or conventional fingerprints (see the Logistic regression section for further details). L1LOG-tensor and L2LOG-tensor use tensor-fingerprints represented by our space-efficient algorithm. L1LOG-concat and L2LOG-concat use previous concatenated fingerprints [7] represented by the LIBLINEAR algorithm [27]. L1LOG-LIBLINEAR-tensor is a method [15, 16] which uses the tensor-product fingerprints represented by the LIBLINEAR algorithm [27].

We conducted the following fold cross-validation in a pair-wise manner. We first randomly divided all drug-protein pairs in the gold standard set into five subsets. Next, we considered four of the subsets as a training set and the remaining subset as a test set. We learned a predictive model on the drug-protein pairs in the training set. Finally, we applied the predictive model to the drug-protein pairs in the test set.

We used the receiver operating characteristic curve (ROC curve), which is defined as a plot of true positive rates against false positive rates based on various thresholds, and the precision-recall curve (PR curve), which is defined as a plot of precision (positive predictive value) against recall (sensitivity) based on various thresholds, as evaluation measures for prediction performance.

We computed the area under the ROC curve (AUC score) and the area under the PR curve (AUPR score). The parameters involved in each method (e.g., regularization parameter) were fit with AUC and AUPR as the objective functions.

**Fig. 7:**
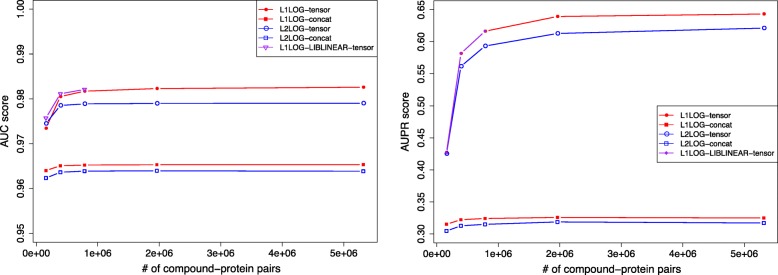
AUC score (left) and AUPR score (right) in pair-wise cross validation

Figure 7 shows the AUC and AUPR scores in the pair-wise cross-validation, where the number of negative pairs in the training set was changed from the same number of positive examples to that of all possible negative examples in the training set. We observed that the prediction accuracy of the models trained with all five methods improved as the number of negative examples in the training set increased. This suggests that using all possible negative examples for learning a predictive model will enhance prediction reliability. L1LOG-tensor performed the best.

L1LOG-LIBLINEAR-tensor did not perform well with an increasing number of negative examples in the training set because of the memory storage problem. The learning process with the LIBLINEAR algorithm consumed all the memory of our machine with 128GB-memory. In contrast, the other four methods with our space-efficient algorithm were able to finish the training process. This suggests that our space-efficient algorithm is more suitable and powerful for learning a predictive model on extremely high-dimensional data.

L1-LOG-tensor and L2LOG-tensor performed better than L1-LOG-concat and L2LOG-concat, which suggests that the tensor-product fingerprint can capture relevant information for drug-protein interaction prediction. On the other hand, the concatenated fingerprint cannot capture enough information, even though calculation is faster.

Table 5 shows the AUC score, AUPR score, training time, and consumed memory in the pair-wise cross-validation. L1LOG-tensor and L2LOG-tensor consumed 24 GB for learning predictive models on all possible drugprotein pairs, which suggests their applicability for largescale drug-protein interaction prediction. They also took about 24 hours, which can be considered reasonable on a practical level, though they were slower than L1LOG-concat and L2LOG-concat.

**Table 5:** AUC score, AUPR score, training time in seconds, and consumed memory in megabytes in the pair-wise cross validation experiments

Method	AUC score	AUPR score	Training time (sec)	Memory (MB)
L1LOG-tensor	0.982±0.000	0.643±0.001	85,211	24,079
L1LOG-concat	0.965±0.000	0.324±0.000	9323	177
L2LOG-tensor	0.979±0.000	0.621±0.000	82,853	24,079
L2LOG-concat	0.963±0.000	0.317±0.000	9129	177
L1LOG-LIBLINEAR-tensor	−	−	−	>131,072

**Fig. 8:**
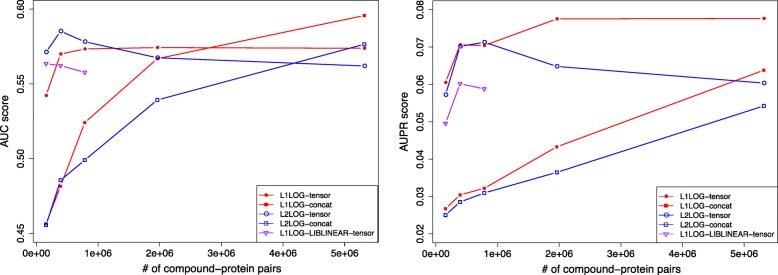
AUC score (left) and AUPR score (right) in block-wise cross validation

In the pair-wise cross-validation, drugs and proteins in test pairs often overlap with those in the training set. We conducted a different 5-fold cross-validation to avoid the overlap of drugs and proteins in test pairs between those in the training set, which we call “block-wise cross-validation”. The results of this block-wise cross-validation are shown in Fig. 8 and Table 6. The same tendency in the pair-wise cross-validation was also seen in the block-wise cross-validation. However, the AUC and AUPR scores in the block-wise cross-validation were much lower than those in the pair-wise cross validation. The results indicate that predictions of unknown interactions for new drug candidates (without known targets) and orphan proteins (without known ligands) are much more difficult than detecting missing interactions between drugs of known targets and proteins of known ligands in practice.

**Table 6:** AUC score, AUPR score, training time in seconds, and consumed memory in megabytes in the block-wise cross validation experiments

Method	AUC score	AUPR score	Training time (sec)	Memory (MB)
L1LOG-tensor	0.574±0.056	0.068±0.002	71,353	19,482
L1LOG-concat	0.596±0.058	0.064±0.002	8323	175
L2LOG-tensor	0.562±0.059	0.060±0.019	70,253	19,482
L2LOG-concat	0.577±0.069	0.054±0.019	8010	175
L1LOG-LIBLINEAR-tensor	−	−	−	>131,072

**Fig. 9:**
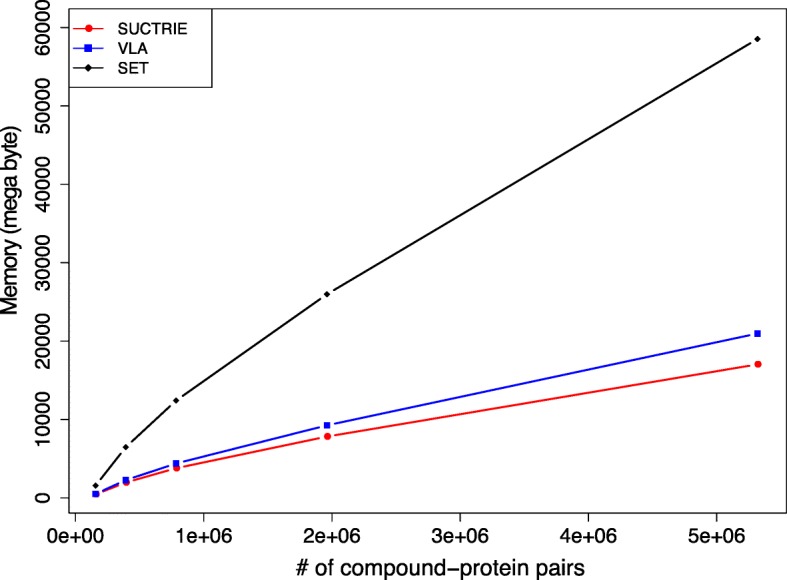
Comparison of consumed memory between different fingerprint representations: SUCTRIE, VLA and SET

Finally, we tested SUCTRIE, VLA, and SET on their space-efficiencies of fingerprint representations. Note that SET is a standard representation, and SUCTRIE and VLA are those constructed with our proposed method. Figure 9 shows a plot of the consumed memory against the number of fingerprints. SET is known to use a large amount of memory for storing all possible fingerprints. In fact, it consumed 57GB for storing all possible drugprotein pairs in our dataset, which limits its practical usage. In contrast, our proposed representations SUCTREE and VLA are more space-efficient than SET. The consumed memory of SUCTREE was slightly smaller than that of VLA. SUCTREE and VLA consumed 16 and 20 GB, respectively, for storing all possible drug-protein pairs, Suggesting the usefulness of our SUCTREE and VLA. In fact, we were not able to conduct all the analyses for this study without SUCTRIE.

## Conclusions

We proposed a novel method of extracting the underlying features characterizing overall drug-protein interactions, which we call “drug-protein interaction signatures”. We extracted a set of drug-protein interaction signatures consisting of the associations between drug chemical substructures, adverse drug reactions, protein domains, biological pathways, and pathway modules, and argued that the extracted drug-protein interaction signatures were biologically meaningful. Our proposed method is original in that the space-efficient representations for high-dimensional fingerprints of drug-protein pairs, in the characterization of a large-scale drug-protein interaction network with various features in an integrative framework, and in the interpretability for the extracted feature associations.

Our proposed method will be useful for various applications in drug discovery. A limitation of the method is that it cannot extract the associations between different attributes of drugs or proteins. For example, it cannot detect the associations between drug-chemical substructures and adverse drug reactions or the associations between protein domains and biological pathways. Extension of the method for analyzing such more complicated features is an important future work.

## Abbreviations

ADRs: Adverse drug reactions
AERS: Adverse event reporting system
AUC score: area under the ROC curve
AUPR score: area under the PR curve FDA: Food and drug administration
KCF-S: KEGG chemical function and substructures
KEGG: Kyoto encyclopedia of genes and genomes
L-BFGS: Limited-memory quasi-newton
OWL-QN: Orthant-wise limited-memory quasi-newton
PR: Precision recall
ROC curve: Receiver operating characteristic curve
